# The Role of Oxidative Stress in Infertility

**DOI:** 10.3390/jpm13081264

**Published:** 2023-08-15

**Authors:** Ioana Zaha, Mariana Muresan, Camelia Tulcan, Anca Huniadi, Petronela Naghi, Mircea Sandor, Roberta Tripon, Cristina Gaspar, Major Klaudia-Melinda, Liliana Sachelarie, Liana Stefan

**Affiliations:** 1 Calla—Infertility Diagnostic and Treatment Center, Constantin A. Rosetti Street, 410103 Oradea, Romania; drzahaioana@gmail.com (I.Z.); petronelanaghi@gmail.com (P.N.); lianaantal@gmail.com (L.S.); 2 Faculty of Medicine and Pharmacy, University of Oradea, 1st December Square 10, 410073 Oradea, Romania; marianamur2002@yahoo.com (M.M.); drims75@gmail.com (M.S.); 3 Faculty of Engineering and Applied Technologies, University of Life Sciences “King Michael I of Romania”, 300645 Timisoara, Romania; tulcancamelia@gmail.com (C.T.); triponroberta97@gmail.com (R.T.); cristina.gaspar@usab-tm.ro (C.G.); 4 ULST Research Institute for Biosecurity and Bioengineering, 300645 Timisoara, Romania; 5 Pelican Clinical Hospital, Corneliu Coposu Street 2, 410450 Oradea, Romania; 6 Szabolcs-Szatmar Bereg County Hospital and University Centre, Jose Andras Hospital, 4400 Nyireghyhaza, Hungary; claudia_major1990@yahoo.com; 7 Department of Prelinical Discipline, Apollonia University, 700511 Iasi, Romania

**Keywords:** follicular fluid, oxidative stress, antioxidant capacity, IVF, serum

## Abstract

(1) Background: Oxidative stress markers in the follicular fluid and serum of the patient with IVF results (number of fertilized oocytes, number of embryos, cumulative pregnancy rate) are important in establishing the causes of infertility. (2) Methods: 42 patients were enrolled in the study over the duration of 24 months and were divided into two groups: the study group divided into the female etiology for infertility as a tubal factor, diminished ovarian reserve, endometriosis, and unexplained infertility, and the control group consisting of the male factor, excluding the sperm donors and with no female factor cause. On the day of ovarian puncture in IVF, follicular fluid and serum were collected from the patients. (3) Results: Malondialdehyde from the follicular fluid was higher in the control group. The total antioxidant capacity in the follicular fluid is positively correlated with the pregnancy rate. There is no statistically significant difference in the oxidative stress markers from serum in both groups. (4) Conclusions: The capacity of the follicular fluid environment to contain the reactive oxygen species (ROS) leads to a higher pregnancy rate in the control group.

## 1. Introduction

Oxidative stress represents the imbalance between the level of oxidation products and the body’s antioxidant capacity. It is at the origin of various pathologies, with a high incidence in modern medicine [1]. Oxidative stress in follicular fluid refers to an imbalance between the productions of reactive oxygen species (ROS) and the ability of the follicular fluid to detoxify these species in the fluid surrounding the developing egg in the ovary. Severe oxidative stress in follicular fluid has been associated with decreased oocyte quality and decreased fertilization rates, which may be due to DNA damage in the egg, changes in the follicular microenvironment, and the decreased availability of antioxidants. This can ultimately lead to decreased pregnancy rates. It is important to note that oxidative stress is just one of many conditions that can affect oocyte and follicle development, and further research is needed to fully understand the relationship between oxidative stress and fertility outcomes [1,2].

In vitro fertilization (IVF) has a known limited success rate and any factors that can be improved are worthy of research [2]. Still, only a small number of oocytes develop into viable embryos that are ready for transfer [3]. The easy way to retrieve follicular fluid during an IVF cycle ovarian puncture makes this medium suitable for examination. It is the environment in which oocyte development takes place, and its composition may also affect oocyte fertilization and embryo quality [4]. In this complex environment of oocyte maturation, oxidative stress could potentially have an adverse effect on the outcome. As a microenvironment, imbalances in follicular fluid affect the deoxyribonucleic acid (DNA) and the cell membrane, lead to poor-quality oocytes and lower the fertilization potential [5]. The follicular fluid compounds consist of steroid hormones, cytokines, growth factors, antioxidants, and vasoregulatory molecules, but the key predictors in inflammation and embryogenesis remain unknown [6,7].

Normal physiological ROS are essential to follicular development and oocyte maturation [7]. In the early stages, the embryo switches from oxidative phosphorylation in the fallopian tube to glycolysis, with high glucose levels in the uterus at implantation in an almost anoxic environment [8]. After placentation, this is reversed and the oxidative phosphorylation increases, with an increase in oxygen levels for cell growth [9].

In the ovary, oxidative stress effects occur in correlation with endometriosis or polycystic ovarian syndrome; the oocyte mitochondrial function, DNA fragmentation and fertilization are impaired. Alterations in the mitochondrial function mean that the development of the oocyte and oocyte maturation are not at peak levels. 

During the IVF process, patients go through a tremendous amount of stress; additionally, some of the causes of infertility come with a pro-inflammatory state: polycystic ovarian syndrome (PCOS), endometriosis, tubal factor, etc. To evaluate the means and degree of their implication in infertility, in addition to the follicular fluid values, the serum values could be related to the pathophysiological pathways [7,8]. 

Reactive oxygen (ROS) produced under oxidative stress, such as malondialdehyde (MDA), protein carbonyl (PC), glutathione disulfide (GSSG), has a damaging effect, and the organism needs defense mechanisms such as non-enzymatic molecules: glutathione (GSH) and enzymatic molecules, such as as superoxide dismutase (SOD), plasma glutathione peroxidase (GSH-Px) and total antioxidant capacity (TAC) [10]. 

**Figure 1:**
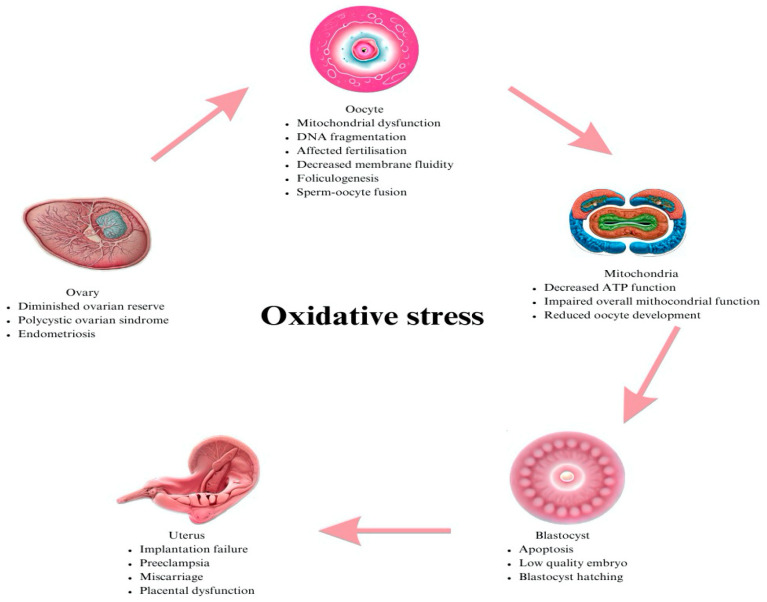
Role of ROS in follicular fluid environment influencing pregnancy rate.

The role of ROS in female infertility in the literature is controversial, there is a paucity of literature, and further investigation is required, as its implications in male infertility were studied more often in the past [7]. Figure 1 shows the detrimental effects of oxidative stress on the female reproductive system. 

A certain amount of oxidative stress is required in early embryo development: spermoocyte fusion, embryo cleavage or blastocyst hatching. The mitochondria generate the oxidative phosphorylation and, therefore, the production of reactive oxygen species in the embryo. The main oxidative stress marker is superoxide dismutase and the counterpart to the non-enzymatic antioxidant is glutathione. In the ovary, oxidative stress effects occur in correlation with endometriosis or polycystic ovarian syndrome; in the oocyte mitochondrial function, DNA fragmentation and fertilization are impaired. Alterations in the mitochondrial function mean that the development of the oocyte and oocyte maturation are not at peak levels. Reactive oxygen species can lead to low-quality embryos and a sequentially low implantation rate, miscarriage, and placental anomalies.

Unexplained infertility affects an important percentage of the IVF population. As well as other pathologies, it could have oxidative stress as an underlying factor. Diminished-ovarian-reserve patients could have low hormone production in the granulosa cells caused by the environmental ROS in follicular fluid, and possibly in patient serum [11,12,13,14].

Polycystic ovarian syndrome (PCOS) is a major cause of infertility and is associated with metabolic syndrome and insulin resistance. Insulin resistance causes lipid peroxidation and, with the hyperglycemia, results in increased oxidative stress that affects steroidogenesis and follicular development [7,15]. PCOS is now recognized by the American Association for the Study of Liver Diseases as a risk factor for nonalcoholic fatty liver disease (NAFLD) [16,17]. Metabolic-associated fatty liver disease (MAFLD) is frequently associated with females with a higher risk of cardiovascular disease and obesity [18].

Body mass index (BMI) is a key indicator when measuring body weight, and based on data from European and American populations, the World Health Organization (WHO) established an international standard, where the normal BMI range is 18.5–24.9 kg/m^2^, a BMI of 25–29.9 kg/m^2^ indicates overweight, and a BMI  ≥  30 kg/m^2^ indicates obesity [16]. Obesity is a risk factor for menstrual disorders and ovulation disorders in women, impairs sperm function and increases the risk of erectile dysfunction in men, thereby reducing fertility [19,20]. Overweight/obesity does not influence pregnancy success; however, it affects the fertilization rate and embryo number [19].

Increased ROS levels in endometriosis are not only a consequence of the chronic inflammation that characterizes this disease but are also caused by ROS detoxification pathway dysregulation [21,22]. Both an upturn in hydrogen peroxide production by mitochondrial superoxide dismutase activity and a drop in catalase activity were found to elevate ROS levels in endometriotic lesions [22]. Oxidative stress may help to promote angiogenesis in ectopic endometrial implants by increasing vascular endothelial growth factor production [23,24].

Oxidative stress biomarkers did not correlate with assisted reproductive technique outcomes, including fertilization, cleavage, or pregnancy rate, in tubal factor infertility [25].

The objective of this study was to measure the values of the oxidative stress markers in infertile patients during the IVF cycle from the follicular fluid and serum and to correlate them with the outcome of the procedure.

The primary outcome was to establish the correlation between the oxidative stress markers (malondialdehyde (MDA), total antioxidant capacity (TAC), superoxide dismutase (SOD), protein carbonyl (PC), plasma glutathione peroxidase (GSH-Px), glutathione (GSH) and glutathione disulfide (GSSG)) and the ovarian response, quantified by the number of oocytes retrieved by ovarian puncture, number of mature oocytes, fertilization rate, number of embryos, blastocyst rate, pregnancy rate and live birth rate (LBR), as well as the cause of infertility.

The secondary outcome was to determine the correlation between maternal age, ovarian reserve (values of anti-Mullerian hormone in ng/mL), HOMA-IR value ((insulin (µU/mL) x glucose (mg/dL))/405) and body mass index (BMI), and the values of oxidative stress markers (malondialdehyde (MDA), total antioxidant capacity (TAC), superoxide dismutase (SOD), protein carbonyl (PC), plasma glutathione peroxidase (GSH-Px), glutathione (GSH) and glutathione disulfide (GSSG)) in the ovarian stimulation outcome.

## 2. Materials and Methods

### 2.1. Patients

In this prospective unicentric pilot study, the research team collected follicular fluid and serum blood sample from the female patients on the day of ovarian puncture during the intracytoplasmic sperm injection (IVF-ICSI) protocol. At first, 82 patients were taken into consideration. After discussing and explaining the procedure, 13 did not sign the accord. After preliminary results, 17 patients needed oocyte and/or sperm donors, and 10 patients had their blood sample or the follicular fluid tampered with, and could not be included. The remaining 42 patients were enrolled in the study over the duration of 24 months from 14 February 2021 until 14 February 2023.

This study had two groups: the control group consisted of male factor infertility and the study group consisted of female factor infertility. The study group is divided into five subgroups based on the ethology of female infertility: tubal factor diminished ovarian reserve, endometriosis, PCOS, and unexplained infertility. The control group consisted of couples for whom the main cause of infertility was the male factor, excluding sperm donors, and there was no female factor cause.

Inclusion Criteria: all patients have an infertility diagnosis and an ovarian puncture and embryo transfer; all patients have a hysteroscopy, are negative for chronic endometritis, and had an ICSI procedure during IVF.

Exclusion Criteria: patients that need donors, patients that refuse to be enrolled in the study or patients with contaminated follicular fluid or serum samples.

The investigation of both partners was completed before enrollment into the study. The basic investigations were semen analysis, tubal patency analysis by hydrosonography or hysterosalpingography, ovarian reserve by antral follicle count and anti-Mullerian hormone levels (AMH). The female patients were also tested for chronic endometritis and uterine cavity by a hysteroscopy prior to embryo transfer. After these investigations, if no underlying pathology was determined, the cause was unexplained infertility, and patients were included in the study group.

This study was approved by the ethics committee of CALLA Center IVF on 14 February 2021 with number 452 and was registered on clinicaltrials.gov on 10 October 2022 with ClinicalTrials.gov Identifier: NCT05575739.

### 2.2. Sample Collection

On the day of oocyte recovery, during IVF protocols, the serum was collected within one hour before or after oocyte recovery. The follicular fluid obtained by aspiration during oocyte recovery from every follicle was collected without blood contamination (contaminated probes were not analyzed and were discarded). The samples were processed by centrifuge and approximately 1 mL of the supernatant was frozen and stored at −80 °C until assay for each marker per patients from follicular fluid, as well as serum from each patient. Every probe was carefully marked with the ID number allocated to the patient and recorded in the database.

### 2.3. Ovarian Stimulation Protocol

An infertility diagnosis, a basal ultrasound and blood sample analysis were carried out on day 2 of the menstrual cycle following the start of the stimulation protocol for each patient. The blood sample consists of estradiol, follicle-stimulating hormone (FSH), luteinizing hormone (LH), and progesterone, and the ultrasound consists of an evaluation of antral follicle count. The stimulation protocol was a short antagonist protocol with doses of recombinant follitropin alfa (Bemfola^®^, Gedeon-Richter, Budapest, Hungary) and HMG (Human menopausal gonadotropin) (Menopur^®^, Ferring, Singapore) individualized for each patient given the ovarian reserve, age, AFC and BMI. After 5 days of stimulation, another blood sample and ultrasound were carried out to search for an increase in estradiol (E2) and follicle average. The administration of the gonadotropin-releasing hormone (GnRH) antagonist (Cetrotide^®^ 0.25 mg, Merck KGaA, Darmstadt, Germany) was given from day 6 of stimulation according to E2 levels (over 300 pg/mL) and follicle average (13 mm), measured by the transvaginal scan. The dose of gonadotropin was adjusted if needed. From day 11 to day 13 of stimulation, patients were given the ovulation trigger (Ovitrelle 250 micrograms/0.5 mL, Merck KGaA) if the two leading follicles measured >17 mm. After 34–36 h, the trigger of the oocyte retrieval was performed by ultrasound guidance. IVF-ICSI was performed approximately 2–4 h after oocyte retrieval. Fertilization was noted if two pronuclei were present at 17–20 h after ICSI. Fresh embryo transfer was performed transcervically under ultrasound guidance in cases where all the criteria were met (progesterone level on the day of trigger lower than 1.2 ng/mL and endometrial thickness of at least 7 mm) on day 5 after ICSI, or all embryos were frozen and a frozen embryo transfer (FET) was carried out in the next cycle. For embryo transfer, the luteal phase support was provided by progestin (Utrogestan 1200 mg, Besins International, Paris, France). Pregnancy was diagnosed by serum levels of beta human chorionic gonadotropin (β-hCG > 20 IU/L) and confirmed with a transvaginal ultrasound with a gestational sac and cardiac activity of the embryo (ongoing pregnancy).

### 2.4. Measurement of Oxidative Stress Markers

An evaluation of oxidative stress marker levels was performed in the Antioxidant Systems Research Laboratory, Research Institute for Biosecurity and Bioengineering from University of Life Sciences “King Michael” Timisoara. All samples were examined in triplicates. The oxidative stress markers malondialdehyde (MDA), total antioxidant capacity (TAC), superoxide dismutase (SOD), protein carbonyl (PC), plasma glutathione peroxidase (GSH-Px), glutathione (GSH) and glutathione disulfide (GSSG) had individual reagents in their own kits by Shanghai Coon Koon Biotech Co. (Shanghai, China) and were determined according to the kit instructions. The test was based on the biotin double-antibody technology enzyme-linked immunosorbent assay (ELISA). A sample (10 μL) was added to the pre-coated objective antibody and (100 μL) Horse Radish Peroxidase (HRP) streptavidin was added to the immune complex. This was shaken and mixed for 60 min at 37 °C incubation and the enzyme was washed with concentration (20×) with distilled water. For color development, 50 μL chromogen solution A was added to each well; then, 50 μL chromogen solution B was added to each well and mixed before incubation for 15 min at 37 °C, away from light, for color development. After 50 μL Stop Solution was added to each well to stop the reaction, the blue color changed into yellow, which is correlated with the concentration of malondialdehyde. According to the measurements of the absorbance (OD) value of samples, the concentration of the corresponding sample was calculated. The standard curve was generated by plotting the average O.D. (450 nm) obtained for each of the six standard concentrations on the vertical (Y) axis versus the corresponding concentration on the horizontal (X) axis. For each sample, the O.D. value was first located on the Y-axis before a horizontal line was extended to the standard curve. At the point of intersection, avertical line was drawn to the X-axis and the corresponding concentration was read. If specimens generated values higher than the highest standard, the specimens were diluted and the assay repeated.

### 2.5. Statistical Analysis

The data were statistically analyzed using SPSS 26. Nonparametric Mann–Whitney U tests were used for group comparisons with statistical significance of *p* < 0.01. Spearman’s correlation analysis was used for correlations. *p* < 0.05 was accepted as statistically significant. Oxidative stress markers were the primary outcome measure.

**Figure 2:**
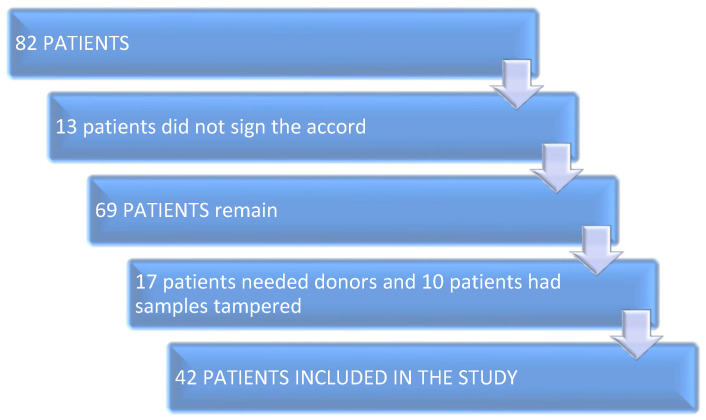
Work flow diagram.

The separate stages of the research are given in Figure 2.

## 3. Results

### 3.1. Outcomes in Both Groups Regarding the Parameters Studied

There was no observed difference between the two groups related to age, homeostasis model assessment and fertilization rate (%), (*p* > 0.001). Regarding anti-Mullerian hormone, body mass index, number of oocytes collected, number of fertilized ovules and pregnancy rate (%), there is a significant difference between the groups (*p* < 0.001), as shown in Table 1.

AMH is a valuable predictor of IVF success. As shown in Table 1, in the control group in which the cause of infertility was the male factor, the ovarian reserve was over the value of 1.5 ng/mL and the results regarding the number of retrieved oocytes were higher than the study group that included the diminished ovarian reserve subgroup. In the control group, the BMI average was higher, and this was an incidental finding because no female patients were selected in this group. The control group subsequently had a higher number of fertilized oocytes due to the number of oocytes that were retrieved, and this higher number of fertilized oocytes was found despite the masculine factor that was determined to be the cause. The cumulative pregnancy rate was higher in the control group and is directly associated with the AMH and number of fertilized oocytes. Even if there was no significant statistical significance, the maternal age was more advanced in the study group, as this was the group that included the diminished ovarian reserve.

The basic characteristics of the patient study subgroups are presented in Table 2.

### 3.2. Comparative Oxidative Stress in Control and Study Group

When the groups were compared in terms of the oxidative stress (follicular fluid) parameters shown in Table 3, statistically significant differences were found in GSH (ug/mL) free glutathione levels (*p* = 0.0001); however, no significant difference was found between the groups in the rest of the parameters (*p* > 0.001).

When the groups were compared in terms of the oxidative stress (serum) parameters shown in Table 4, no significant difference was found between the groups (*p* > 0.001).

### 3.3. Corelation between Oxidative Stress and IVF Parametres

#### 3.3.1. Control Group

The correlations between IVF parameters and oxidative stress markers (follicular fluid) for the control group are shown in Table 5. A positive correlation was seen between MDA (nmol/mL—from follicular fluid) and cumulative pregnancy rate (%), (*p* = 0.009). There is also a correlation between TAC (U/mL—from follicular fluid) and cumulative pregnancy rate (%), (*p* = 0.006). MDA in follicular fluid is correlated with a higher oxidative stress in the environment. This, to some degree, is beneficial to the production of steroid hormones, but detrimental in high amounts. The total antioxidant capacity in the follicular fluid is positively correlated with the pregnancy rate, a marker of the follicular fluid’s capacity to maintain homeostasis between the antioxidants and ROS.

Regarding the oxidative stress markers in the serum, it is observed that there is no statistically significant correlation in both the control group and the study group.

#### 3.3.2. Study Group

**Figure 3:**
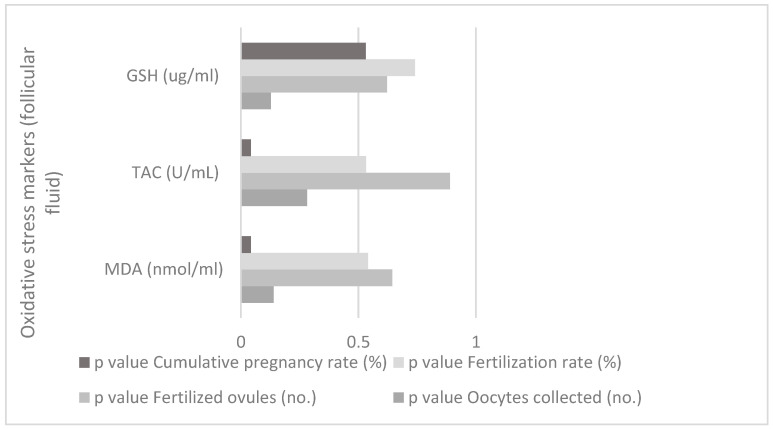
Correlation between oxidative stress markers (follicular fluid) and IVF parameters for the study group.

There is a correlation between oxidative stress markers (follicular fluid) and IVF parameters for study group between MDA (nmol/mL—from follicular fluid) and cumulative pregnancy rate (%), (*p* = 0.042, *p* < 0.05), and between TAC (U/mL—from follicular fluid) and cumulative pregnancy rate (%), (*p* = 0.042, *p* < 0.05), as shown in Figure 3.

**Figure 4:**
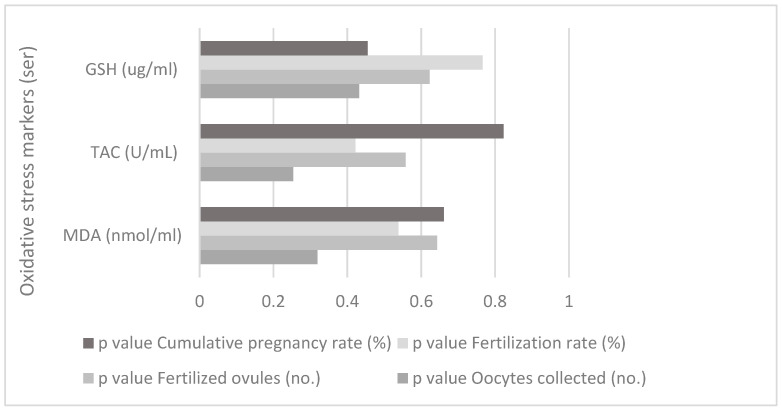
Correlation between oxidative stress markers (serum) and IVF parameters for the study group.

There is no correlation between the oxidative stress markers from ser and IVF parameters for the study group, as shown in Figure 4.

## 4. Discussion

The embryo development has a direct correlation with the oocyte and the fertilization of the oocyte. Follicular fluid is the environment in which the oocyte is found, along with other components that have an important role in the maturation of the oocyte [26]. Over the past decade, the role of oxidative stress in IVF outcome has not been unanimously accepted, with contradictory results and no clear correlation between the pro-inflammatory ROS and the natural antioxidant barrier.

In our study, there was no significant difference between the control and study group in women’s age. This could be because the groups had similar age gaps and there was a non-biased selection of patients. Age is an important parameter in IVF and women should be counseled about the age-related risk of infertility and referred to a fertility center as early as possible [27]. HOMA had no impact on the outcome in both groups but the BMI made a difference in terms of the number of oocytes, fertilization rate and cumulative pregnancy rate. A more optimal HOMA and BMI will lead to better IVF results [28]. In the male control group, we should consider the fact that the testes are much more susceptable to oxidative injury due to their poor antioxidant defense mechanism [29].

The AMH value had an important role in the IVF outcome for the control group, as the study group contained the diminished-ovarian-reserve patients (*p* = 0.0103). This led to a higher number of oocytes being collected and fertilized and a higher cumulative pregnancy rate in the control group. Age, AMH and the number of oocytes obtained are independent factors influencing clinical pregnancy [30].

According to our results, MDA (nmol/mL) was higher in the study and control group with a higher cumulative pregnancy rate (*p* = 0.421). This is like the findings found in other studies, where pregnant women had a higher peroxidation level [31]. Lipid peroxidation or the reaction of oxygen with unsaturated lipids has malondialdehyde as a secondary product and appears to be the most mutagenic product but not the most toxic [31]. In the presence of endometriosis, high levels of MDA were detected in FF in a new study [32,33]. Appasamy M et al. found that ROS in follicular fluid had a positive impact on the pregnancy rate during IVF [34]. This finding could mean that the amount of oxidative stress required during the oocyte maturation could be beneficial to the embryo development process. Oral O et al. revealed that there was no significant relationship between follicular fluid malondialdehyde and fertilization rate or no correlation between IVF outcome and oxidative stress markers [34,35]. Comparing pregnant women to non-pregnant women, the results show that the pregnant women had higher lipid peroxidation [30] but other studies show that ROS is negatively correlated with embryo quality and pregnancy [30,36].

In this study, the GSH levels (ug/mL) were higher in the study group in women with various causes of infertility. The natural mechanisms aim to counteract oxidative stress rises in the follicular fluid protect and maintain a balance during oocyte maturation. In some studies, GSH is positively linked to the formation of pronucleus and improves fertilization and embryo quality in culture media [14,37]. The high levels of GSH concentration found in follicular fluid have an important antioxidant protective role for oocyte maturation [38,39]. The literature on this topic is sparse. Ozkaya MO et al. studied the effect of multivitamin and mineral supplementation on GSH levels in follicular fluid and serum in women with unexplained infertility in 2010, but no correlation with the IVF outcome was found [40]. The seven types of oxidative stress markers, both anti-oxidative and pro-oxidative, were tested from the follicular fluid during oocyte retrieval. In the study group that contained the various causes of female infertility, the value of one of the most important natural enzymatic antioxidants, free glutathione, was higher and had statistical significance. The study group in this research included PCOS and other proinflammatory pathologies. The GSH-dependent antioxidant system acts more efficiently in the advanced maturation stages of oocytes and embryos, and the antioxidant quantities in the FF can be potential predictors of oocyte and embryo quality, as one study showed in PCOS women [41].

In our study, the total antioxidant capacity (U/mL) was correlated with a higher pregnancy rate in both the control and study group, meaning that both groups had a good antioxidant response in the medium of oocyte maturation, leading to mature oocytes and good-quality embryos that are capable of implantation. The cumulative pregnancy rate is higher in the study group with high TAC values in follicular fluid, meaning the study group had a better defense mechanism against the ROS. The MDA was higher in the follicular fluid in the study group with a better cumulative pregnancy rate as well as high levels of TAC, which could explain the fac that the defense mechanism works and the medium in which the oocytes develop has good homeostasis if there are no other influences.

The oxidative stress markers studied in the patient serum at the moment of oocyte retrieval, along with the follicular fluid, did not have major implications in the IVF outcome. Serum OS markers were higher in the endometriosis groups, suggesting an impact of pituitary downregulation with GnRH and ovarian stimulation on the oxidative balance of these patients, especially on the day of oocyte retrieval [38,39].

A fair amount of oxidative stress but not an excessive amount is vital in embryo development and the embryo itself generates ROS to implant [42].

ROS plays an important role in women’s fertility pathways from the beginning to pregnancy. The ratio between pro-oxidants and antioxidants represents the overall oxidative stress milieu and seems to be a significantly better predictor of achieving pregnancy than the ROS level or antioxidant level alone [43].

The supplementation of vitamins and antioxidants (L-arginine, vitamin E, myo-inositol, D-chiro-inositol, carnitine, selenium, vitamin B complex, vitamin C, vitamin D + calcium, CoQ10 (Coenzyme Q10), and omega-3 polyunsaturated fatty acids) was not proven to have a beneficial role in IVF [44]. An association was found between antioxidant use and the development of clinical pregnancy rates among women with PCOS [41,42].

Increased ROS levels in the embryo can cause mitochondrial changes in more vulnerable DNA and can cause metabolic malfunctions and disrupt the development of the embryo, possibly leading to apoptosis [44]. In recent studies, both antioxidant supplementation and accurate embryo culture, as well as safe laboratory conditions, could be the key to obtaining quality embryos that lead to higher pregnancy rates during IVF cycles [43]. Identifying the hormonal changes and the action of antioxidants may help to develop new therapeutic strategies for hormonal-imbalance-related disorders [44].

Further studies are needed to clearly establish the exact implications of oxidative stress in human reproduction. The limitations of the study could be the lack of a multicentric study validation and the niche of infertility, as well as the fact that it is unknown how ROS affects or is abolished in women that do not struggle with infertility. Furthermore, the small number of enrolled patients could be a study limitation, as well as the niche subject of interest, with it not being known how this applies to the general population without an infertility diagnosis.

## 5. Conclusions

Oxidative stress in human reproduction is just a part of many factors that contribute to the success rate of pregnancy. IVF cannot control all the factors, but we try to study and adjust everything that can be modified. In IVF cycles, the number of quality oocytes does not depend on apoptosis or oxidative stress alone. To further evaluate the relationship between the oxidative stress markers, additional studies with large and homogenous patient groups are needed, along with a standardization of the used protocol. The microenvironment of the follicular fluid could predict the development of mature oocytes and thus the top-quality embryos. The aim is to obtain a range of reference markers that can help determine whether supplementation with antioxidants is necessary, or denote the minimum maintenance value of pro-inflammatory markers. In many IVF centers, embryos are selected according to their morphological features, but follicular fluid pro-inflammatory cytokines and oxidative stress markers that can improve the outcomes could also be studied. The findings in our study suggest the influence of the microenvironment on the oocyte due to pro-inflammatory imbalances, and that antioxidants have an impact on embryo development and embryo implantation.

## Figures and Tables

**Table 1:** Variables in control and study groups.

Variables	Control Group (*n* = 17)	Study Group (*n* = 25)	*p* Value
Age	32.82 ± 7.10	36.08 ± 5.16	0.0927
Anti-Mullerian hormone (AMH) ng/mL	2.94 ± 1.99	1.49 ± 1.5	0.0103
Body mass index (BMI)	28.62 ± 4.7	25.48 ± 4.77	0.0412
Homeostasis model assesssment (HOMA)	3.04 ± 1.32	3.24 ± 2.12	0.7282
No. oocytes collected (no.)	14.41 ± 5.75	8.72 ± 6.80	0.0073
No. fertilized ovules (no.)	8.82 ± 4.61	5.12 ± 4.49	0.0132
Fertilization rate (%)	75.49 ± 19.30	74.30 ± 23.90	0.8648
Cumulative pregnancy rate (%)	69.60 ± 44.18	38.00 ± 46.27	0.0327

**Table 2:** Variables in study subgroups.

Variables	Tubal Factor (*n* = 13)	Diminished Ovarian Reserve (*n* = 16)	Endometriosis (*n* = 3)	PCOS (*n* = 8)	Unexplained Infertility (*n* = 5)
Age (years)	31.8 (31.1 ± 3.5)	30.8 (27.4 ± 4.2)	35.8 (34.6 ± 1.1)	34.41 (33.5 ± 1.6)	32.8(32.1 ± 3.5)
Anti-Mullerian hormone (AMH) ng/mL	1.49 ± 1.5	1.23 ± 1.2	1.19 ± 1.3	1.37 ± 1.4	1.29 ± 1.1
Body mass index (BMI)	25.6 ±1.3	26.1 ±7.1	27.4 ±4.1	22.2 ±3.1	21.6 ±2.2
Homeostasis model assesssment (HOMA)	3.24 ± 2.12	3.24 ± 2.12	1.24 ± 2.12	3.34 ± 2.15	3.24 ± 2.12
No. oocytes collected (no.)	5.22 ± 5.80	3.32 ± 3.80	3.72 ± 2.70	8.72 ± 5.80	2.72 ± 3.80
No. fertilized ovules (no.)	2.12 ± 3.49	4.12 ± 3.49	3.12 ± 4.29	6.12 ± 3.49	3.12 ± 2.27
Fertilization rate (%)	47.9 ± 13.80	39.5 ± 23.80	38.9 ± 12.70	39.4 ± 43.30	47.8 ± 11.60
Cumulative pregnancy rate (%)	37.00 ± 36.24	35.00 ± 23.21	38.00 ± 28.23	35.00 ± 12.35	34.00 ± 34.25

**Table 3:** Comparation of oxidative stress between control and study group (follicular fluid).

	Control Group (*n* = 17)	Study Group (*n* = 25)	*p* Value
MDA (nmol/mL)	147.86 ± 40.69	148.72 ± 26.95	0.9345
(U/mL) total antioxidant capacity (TAC)	11.83 ± 4.80	12.24 ± 3.63	0.7549
(U/mL) superoxide dismutase (SOD)	44.47 ±5.08	42.61 ± 6.43	0.3252
(pg/mL) carbonylated proteins (PC)	169.60 ± 27.35	173.26 ± 25.97	0.6638
(U/mL) plasma glutathione peroxidase (GSH-Px)	147.49 ± 17.10	141.61 ± 28.03	0.4454
(ug/mL) free glutathione (GSH)	94.70 ± 20.91	141.61 ± 28.03	0.0001
(ng/mL) gluthation disulfit (GSSG)	224.67 ± 20.73	214.12 ± 23.38	0.1411

**Table 4:** Comparation of oxidative stress between control and study group (serum).

	Control Group (*n* = 17)	Study Group (*n* = 25)	*p* Value
MDA (nmol/mL)	136.29 ± 40.15	134.56 ± 33.94	0.8812
TAC (U/mL)	11.47 ± 2.71	11.19 ± 3.28	0.7895
SOD (U/mL)	44.42 ± 8.19	40.40 ± 7.68	0.1129
PC (pg/mL)	168.90 ± 35.18	165.95 ± 23.07	0.7512
GSH-Px (U/mL)	142.68 ± 22.69	132.80 ± 26.18	0.2130
GSH (ug/mL)	90.12 ± 10.60	84.16 ± 12.59	0.1171
GSSG (ng/mL) gluthation disulfit	218.38 ± 33.11	204.09 ± 32.65	0.1742

MDA (nmol/mL) Malondialdehyde; TAC (U/mL) total antioxidant capacity; SOD (U/mL) superoxide dismutase; PC (pg/mL) carbonylated proteins; GSH-Px (U/mL) plasma glutathione peroxidase; GSH (ug/mL) free glutathione; GSSG (ng/mL) glutathione disulfide; *p* < 0.01 was considered statistically significant.

**Table 5:** Correlation between oxidative stress markers (follicular fluid, serum) and IVF parameters for control group.

Oxidative Stress Markers (Follicular Fluid)	Oocytes Collected (no.)	Fertilized Ovules (no.)	Fertilization Rate (%)	Cumulative Pregnancy Rate (%)
MDA (nmol/mL)				
*p* value	0.129	0.944	0.846	0.009
TAC (U/mL)				
*p* value	0.155	0.988	0.846	0.006
GSH (ug/mL)				
*p* value	0.252	0.940	0.387	0.660
Oxidative stress markers (ser)				
MDA (nmol/mL)				
*p* value	0.029	0.833	0.741	0.668
TAC (U/mL)				
*p* value	0.173	0.789	0.532	0.887
GSH (ug/mL)				
*p* value	0.152	0.877	0.267	0.773

MDA (nmol/mL) Malondialdehyde; TAC (U/mL) total antioxidant capacity; GSH (ug/mL) free glutathione; GSSG (ng/mL) glutathione disulfide; *p* < 0.05 was considered statistically significant; Spearman’s correlation.
